# Defense-Related Transcriptional Reprogramming in Vitamin E-Deficient Arabidopsis Mutants Exposed to Contrasting Phosphate Availability

**DOI:** 10.3389/fpls.2017.01396

**Published:** 2017-08-10

**Authors:** Annapurna D. Allu, Bárbara Simancas, Salma Balazadeh, Sergi Munné-Bosch

**Affiliations:** ^1^Institute of Biochemistry and Biology, University of Potsdam Potsdam-Golm, Germany; ^2^Max-Planck-Institut für Molekulare Pflanzenphysiologie Potsdam, Germany; ^3^Department of Evolutionary Biology, Ecology and Environmental Sciences, Faculty of Biology, University of Barcelona Barcelona, Spain

**Keywords:** antioxidants, photosystem II, plastochromanol-8, priming, retrograde signaling, tocochromanols, vitamin E

## Abstract

Vitamin E inhibits the propagation of lipid peroxidation and helps protecting photosystem II from photoinhibition, but little is known about its possible role in plant response to Pi availability. Here, we aimed at examining the effect of vitamin E deficiency in *Arabidopsis thaliana vte* mutants on phytohormone contents and the expression of transcription factors in plants exposed to contrasting Pi availability. Plants were subjected to two doses of Pi, either unprimed (controls) or previously exposed to low Pi (primed). In the wild type, α-tocopherol contents increased significantly in response to repeated periods of low Pi, which was paralleled by increased growth, indicative of a priming effect. This growth-stimulating effect was, however, abolished in *vte* mutants. Hormonal profiling revealed significant effects of Pi availability, priming and genotype on the contents of jasmonates and salicylates; remarkably, *vte* mutants showed enhanced accumulation of both hormones under low Pi. Furthermore, expression profiling of 1,880 transcription factors by qRT-PCR revealed a pronounced effect of priming on the transcript levels of 45 transcription factors mainly associated with growth and stress in wild-type plants in response to low Pi availability; while distinct differences in the transcriptional response were detected in *vte* mutants. We conclude that α-tocopherol plays a major role in the response of plants to Pi availability not only by protecting plants from photo-oxidative stress, but also by exerting a control over growth- and defense-related transcriptional reprogramming and hormonal modulation.

## Introduction

Tocopherol cyclase (VTE1), which is located in plastoglobules (Vidi et al., 2006), is a key enzyme for the biosynthesis of both plastochromanol-8 and vitamin E compounds (Sattler et al., 2003; Szymanska and Kruk, 2010). As the content of plastoglobules is in equilibrium with thylakoid membranes (Austin et al., 2006), both plastochromanol-8 and vitamin E compounds are found in thylakoids. These together fulfill an antioxidant function protecting lipids from the propagation of lipid peroxidation and prevent photosystem II damage, the latter function being performed together with carotenoids (Munné-Bosch and Alegre, 2002; Havaux et al., 2005; Falk and Munné-Bosch, 2010; Zbierzak et al., 2010; Kruk et al., 2014). Apart from this antioxidant function, tocochromanols may play a major role in cellular signaling by influencing redox, hormonal, and sugar regulatory networks, an aspect that has already been shown in key developmental processes such as seed germination (Mène-Saffrané et al., 2010) or leaf senescence (Abbasi et al., 2009), and plant responses to abiotic stresses, including salinity (Abbasi et al., 2007; Cela et al., 2011; Asensi-Fabado et al., 2015), osmotic stress (Abbasi et al., 2007), high light (Munné-Bosch et al., 2007), low temperatures (Maeda et al., 2008), and water deficit (Cela et al., 2011). Although a previous study has shown that vitamin E and inorganic phosphate (Pi) availability exert a complex interplay in the control of longevity in *Arabidopsis thaliana* (Simancas and Munné-Bosch, 2015), nothing is known about the possible influence of vitamin E on plant response to contrasting Pi availability.

The response of plants to several environmental stress factors, including low phosphate availability in soils, involves intricate regulatory networks governed by various signaling molecules. Pi is a major macro element source for plant growth; hence one of the most prominent effects of Pi starvation is reduced plant growth (Marschner, 2012). However, to maintain cellular Pi homeostasis under conditions of Pi starvation, plants have evolved a series of adaptive responses such as limiting Pi consumption and internally adjusting Pi recycling (Sato and Miura, 2011). Several hormones such as abscisic acid (ABA), ethylene, auxin, and cytokinin have been shown to be involved in plant response to varying Pi availability conditions (Franco-Zorrilla et al., 2004; Rubio et al., 2009). Plants coordinate Pi homeostasis with its carbon status and photosynthesis through sophisticated mechanisms and phytohormones play a crucial role in cross-talking the Pi starvation with sugar signaling (Franco-Zorrilla et al., 2004; Wissuwa et al., 2005; Rubio et al., 2009). Cytokinins negatively regulate Pi starvation responses and its content is reduced under Pi starvation (Yang and Finnegan, 2010). Cytokinins are also proposed to be interacting with sugars in Pi starvation signaling (Franco-Zorrilla et al., 2005). Both auxin (auxin-dependent and independent) and ethylene pathways are known to regulate root architecture in response to Pi availability (Franco-Zorrilla et al., 2004; Rouached et al., 2010). Impaired ABA sensitivity (*abi2-1*) or biosynthesis (*aba1*) mutants display reduced Pi-responsive gene expression and anthocyanin accumulation (Trull et al., 1997; Ciereszkoa and Kleczkowsk, 2002). Furthermore, the gibberellin-DELLA signaling pathway plays a role in the regulation of plant stature, root architecture changes, and anthocyanin accumulation under low Pi-conditions involving ubiquitin-mediated protein degradation (Jiang et al., 2007).

Extensive gene expression changes that integrate signals from external and internal factors are indispensable in the execution of evolved intricate adaptive strategies under low Pi conditions and are witnessed by transcriptome analysis in several plant species (Yang and Finnegan, 2010). Transcription factors (TFs) are the major regulators of stress-associated gene expression changes. Several recent studies have identified TFs involved in the regulation of Pi availability-related gene expression changes. PHOSPHATE STARVATION RESPONSE 1 (PHR1), a member of the MYB TF super family was identified in a mutant screen, where the mutants are impaired in Pi-responsive transcript and anthocyanin accumulation (Rubio et al., 2001; Bari et al., 2006). *OsPHR1* and *2*, the two *AtPHR1* orthologs in rice, have been identified to regulate the Pi-deficiency response similar to that in Arabidopsis (Zhou et al., 2008). Further, a Pi-starvation induced TF, MYB62, plays a major role during Pi limitation resulting in changes in root length, root phosphatase activity, and anthocyanin accumulation. The MYB62-mediated Pi starvation response has been proposed to act through the regulation of gibberellin levels (Devaiah et al., 2009; Yang and Finnegan, 2010). Few other TFs that were identified to have a functional role in the Pi starvation response include Arabidopsis ZAT6, bHLH32, PTF1, WRKY75, and rice OsWRKY74, among others (Yi et al., 2005; Chen et al., 2007; Devaiah et al., 2007a,b; Dai et al., 2016). Importantly, these TFs act as nodes in the crosstalk between Pi starvation- and hormone-signaling in regulating the plant response to Pi limitation (Rouached et al., 2010), but only a few TFs have been identified so far.

Despite Pi addition is a common practice to increase yield in cultivated plants and plant response to low Pi has been studied in detail, the underlying mechanisms explaining plant response to reiterated changes in Pi availability has been poorly studied to date. In poplar, it was found that stem cuttings derived from sites with lower Pi availability established worse, irrespective of Pi level after transplantation, which was correlated with differences in DNA methylation (Schönberger et al., 2016). Epigenetic modifications are of high interest to better understand priming or memory effects and may serve as an excellent basis to better exploit Pi resources, an important nutrient that is very likely to become limited in the near future (Herrera-Estrella and López-Arredondo, 2016). In this respect, current genetic resources in the model plant *A. thaliana* can be used to better understand priming effects in plant response to contrasting Pi availability.

Chloroplast-nuclear retrograde signaling regulates gene expression, but its integration with redox and hormonal signaling is still poorly understood (Pfannschmidt and Munné-Bosch, 2013). It has been shown that changes in the vitamin E composition in chloroplasts profoundly alters gene expression in the nucleus, particularly of ethylene-related signaling genes, including *ERF1* (Cela et al., 2011), an essential regulatory hub of ethylene, jasmonic acid, and ABA signaling (Müller and Munné-Bosch, 2015). Recently, transorganellar complementation has also revealed that vitamin E can access the lumen of the endoplasmic reticulum without necessarily involving transporters (Mehrshahi et al., 2013), which opens the possibility of vitamin E directly influencing redox signaling outside chloroplasts.

In the current study, with the aim of getting new insights into the possible retrograde signaling function of vitamin E as an antioxidant in chloroplasts, we examined the response of wild type and vitamin E-deficient *A. thaliana* plants to contrasting levels of Pi availability, including a priming treatment. We aimed at understanding the effect of varying Pi availability on the expression of whole TFs that govern the downstream gene expression changes orchestrating plant growth and response to low Pi conditions. Emphasis was put on the possible effects of vitamin E deficiency on TFs and hormonal profiling.

## Materials and Methods

### Plant Material, Treatments, and Sampling

**FIGURE 1:**
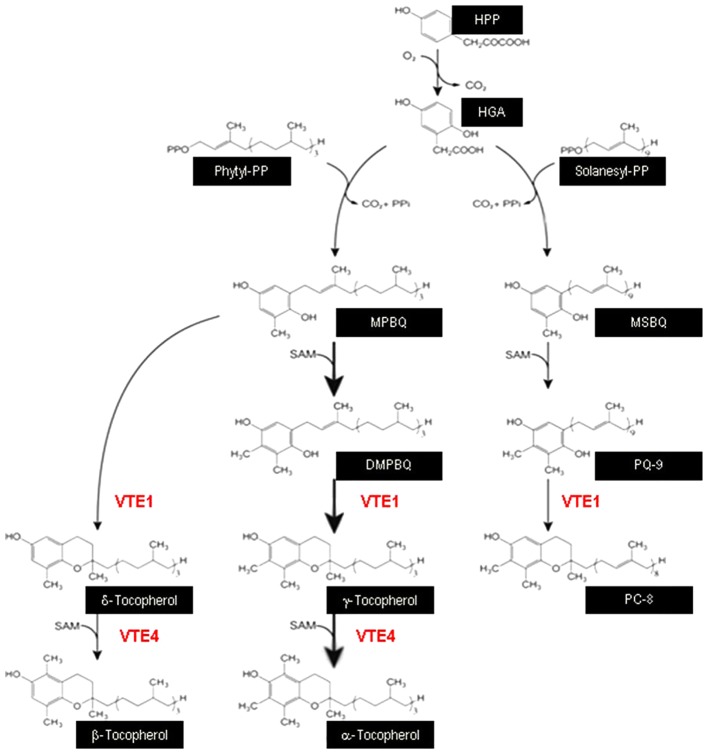
Schematic diagram displaying the central role of VTE1 and VTE4 in the biosynthesis of vitamin E. VTE1, tocopherol cyclase; VTE4, γ-tocopherol methyltransferase; HPP, hydroxyphenylpyruvate; HGA, homogentisic acid; MPBQ, methylphytylbenzoquinol; MSBQ, methylsolanesylbenzoquinol; DMPBQ, dimethylphytylbenzoquinol; PQ-9, plastoquinone-9; PC-8, plastochromanol-8.

Seeds of *A. thaliana* Columbia ecotype (Col-0), and *vte1* (GK_111D07) and *vte4* (SALK_036736) mutants were used in this study. *vte1* and *vte4* mutants have T-DNA insertions in the *VTE1* and *VTE4* genes (Porfirova et al., 2002; Bergmüller et al., 2003), which encode tocopherol cyclase and γ-tocopherol methyltransferase, respectively, so that the *vte1* mutant lacks both α- and γ-tocopherol, and the *vte4* mutant lacks α-tocopherol but accumulates γ-tocopherol (**Figure 1**).

**FIGURE 2:**
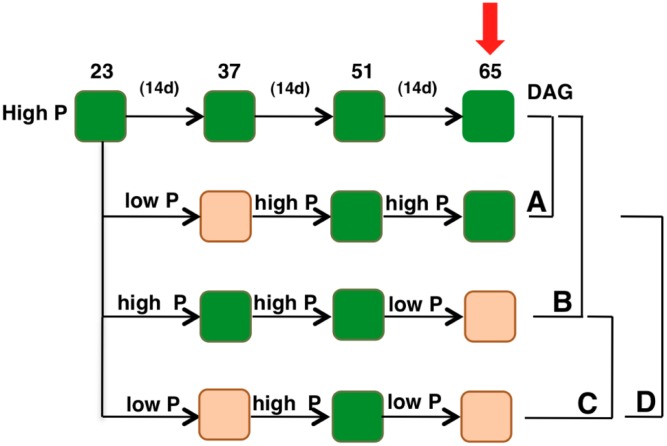
Schematic diagram displaying the experimental plan. All the genotypes were grown with 1 mM potassium phosphate (hereafter called high Pi) for 23 days. Contrasting Pi levels were then supplied to the plants in three different sets. Orange blocks indicate periods during which the plants were supplied with 0.5 mM potassium phosphate (hereafter called low Pi). Arrow indicates the time point when sampling was performed. DAG, days after germination. (A–C) Show comparisons used for data analyses.

**FIGURE 3:**
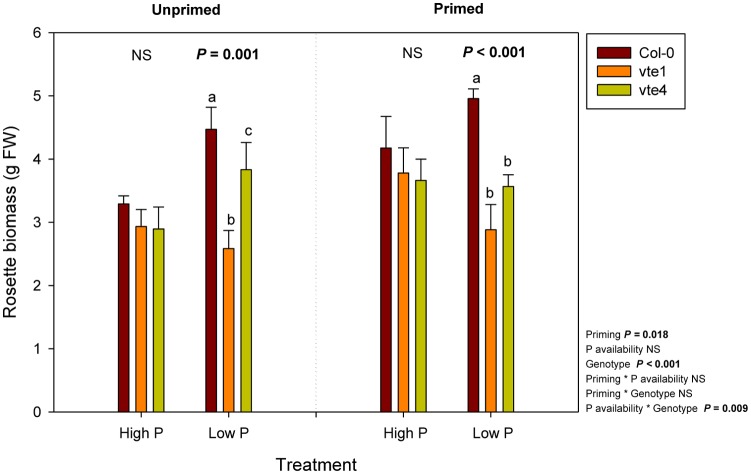
Rosette biomass of vitamin E-deficient (*vte1* and *vte4* mutants) and wild-type plants of *Arabidopsis thaliana* exposed to contrasting Pi availability, including unprimed and primed plants. Data represent the mean ± SE of *n* = 6 individuals. Significant differences between groups were tested by three-way analysis of variance (ANOVA, *P* < 0.05). Different letters significant differences between genotypes at any given treatment (Duncan *post hoc* tests, *P* < 0.05). NS, not significant.

Seeds were cold-stratified and sown in 0.1 L-pots in soil (Einheitserde GS90; Gebrüder Patzer) in a climate-controlled chamber with a 8-h day length provided by fluorescent light at 100 μmol m^-2^ s^-1^, a day/night temperature of 20/16.8°C and a relative humidity of 60/75% (day/night). Prior to treatments, plants were watered every third day during 23 days with high Pi nutrient solution containing 5 mM Ca(NO_3_)_2_, 5 mM KNO_3_, 2 mM MgSO_4_, 1 mM KH2PO_4_, and 5 g/L iron chelate (EDTA FeNa). Next, plants were divided into four sets and were subjected to varying phosphate treatments (**Figure 2**). One set of plants was supplied with the same high Pi nutrient solution (containing 1 mM KH_2_PO_4_) every other day throughout the experiment (high Pi-plants). Contrasting Pi levels were then supplied to the other three set of plants by using 0.5 mM instead of 1 mM KH_2_PO_4_ in the nutrient solution (low Pi). This nutrient solution was supplied with 0.5 mM KCl to compensate for K deficiency. Typically, low Pi (0.1 mM) and high Pi (ranging between 0.5 and 2.5 mM) are used in studies on Pi starvation in *A. thaliana* (Williamson et al., 2001). However, our studies indicated that 0.5 mM Pi is an adequate concentration to induce priming effects ultimately resulting in enhanced growth (**Figure 3**).

Samplings were performed at midday (in the middle of the photoperiod) at the end of treatments. Whole rosettes of six individuals were used to estimate leaf water contents, chlorophyll levels, the *F*_v_/*F*_m_ ratio, and the levels of plastochromanol-8 and vitamin E, as well as the TFs and hormonal profiling. Samples for biochemical and transcriptional analyses were collected, immediately frozen in liquid nitrogen and stored at -80°C until analysis.

### Leaf Water and Nutrient Content, Chlorophyll Level, *F*_v_/*F*_m_ ratio and Lipid Peroxidation

Samples were weighed to estimate the fresh matter (FW), immersed in distilled water at 4°C for 24 h to estimate the turgid matter (TW) and then oven-dried at 80°C to constant weight to estimate the dry matter (DW). Relative water content (RWC) was then calculated as 100 × (FW - DW)/(TW - DW). For analyses of macro- and micronutrients (P, S, Ca, Mg, K, Na, Mn, Zn, Fe, Mo, B, Cu, and Si), dried samples were weighed, digested with HNO_3_, and analyzed by inductively coupled plasma atomic emission spectroscopy (ICP-AES). For pigment analysis, measurements were performed using a SPAD 502 Plus chlorophyll meter. The maximum efficiency of the photosystem II (*F*_v_/*F*_m_ ratio) was determined measuring the chlorophyll fluorescence of leaves by using a pulse-modulated fluorometer (Mini PAM; Walz, Effeltrich, Germany) as described by Genty et al. (1989). The extent of lipid peroxidation was estimated by measuring the levels of malondialdehyde (MDA) in leaves. MDA levels were estimated spectrophotometrically following the thiobarbituric acid-reactive assay considering the effect of potential interfering compounds, as described (Hodges et al., 1999).

### Tocochromanol Contents

For analyses of vitamin E and plastochromanol-8 contents, leaf samples (50 mg) were ground in liquid nitrogen and extracted with cold methanol (v/v) using ultra-sonication. After centrifuging at 8000 rpm for 10 min and 4°C, the supernatant was collected and the pellet re-extracted with the same solvent until it was colorless; then, supernatants were pooled, filtered, and injected into the HPLC. Tocochromanols were separated isocratically on a normal-phase HPLC system using a fluorescent detector as described (Cela et al., 2011). Compounds were identified by co-elution with authentic standards and quantified by using a calibration curve.

### Transcription Factor Profiling

For the large-scale TF (a total of 1,880 TFs) profiling using qRT-PCR, total RNA was extracted from whole rosette leaves. Primer sequences are provided in Supplementary Table 10. Total RNA extraction, synthesis of cDNA, and qRT-PCR were performed as described (Caldana et al., 2007). ACTIN2 was used as reference gene. PCR reactions were run on an ABI PRISM 7900HT sequence detection system (Applied Biosystems Applera), and SYBR Green (Life Technologies) was used for visualizing amplified products.

### GO Enrichment Analysis

Gene ontology (GO) enrichment analysis for the priming specific TFs was performed using PLAZA 3.0 using default settings (Proost et al., 2015).

### Clustering Analysis

Differentially expressed genes were visualized as heatmaps using multiple expression viewer (Mev^1^; Saeed et al., 2003). Cluster analysis for differentially expressed genes was performed with Short Time-series Expression Miner (STEM) software using default settings (Ernst and Bar-Joseph, 2006).

### Hormonal Profiling

For analyses of cytokinins, auxin, gibberellins, ABA, salicylic acid, jasmonates, the ethylene precursor, 1-aminocyclopropane-1-carboxylic acid, and melatonin, leaf samples (50 mg) were ground in liquid nitrogen and extracted with cold methanol:isopropanol:acetic acid (50:49:1, v/v/v) using ultra-sonication. After centrifuging at 8000 rpm for 10 min and 4°C, the supernatant was collected and the pellet re-extracted with the same solvent until it was colorless; then, supernatants were pooled, filtered, and injected into the UHPLC-MS/MS. Phytohormones were separated using an elution gradient on a reverse-phase UHPLC system and quantified using tandem mass spectrometry in multiple reaction monitoring mode as described (Müller and Munné-Bosch, 2011). Recovery rates were calculated for each hormone on every sample by using deuterated compounds.

### Statistical Analysis

Data was analyzed by using three-way and one-way factorial analysis of variance (ANOVA), and by additionally using Duncan *post hoc* tests to analyze for the effects of genotypes at each condition. In all cases, differences were considered significant at a probability level of *P* < 0.05. All statistical tests were carried out using the SPSS 20.0 statistical package.

## Results

### Plant Response to Contrasting Phosphate Availability

In order to understand plant adaptive mechanisms to contrasting Pi availability, plants were grown in a controlled environment initially to attain uniform growth among all the genotypes under study. Later, one set of plants were supplied with the same Pi concentration (hereafter called high Pi), whereas half the concentration (hereafter called low Pi, see “Materials and Methods”) was supplied to plants in three different sets to understand plant response to contrasting Pi availability and priming effects. The first set included plants supplied with low Pi for 2 weeks and then returned to high Pi; the second set included plants grown under high Pi condition and then exposed to low Pi for 2 weeks just prior to samplings. To capture the effects of priming, plants were exposed to low Pi, returned to high Pi condition and then later exposed to second spell of low Pi (**Figure 2**).

**FIGURE 4:**
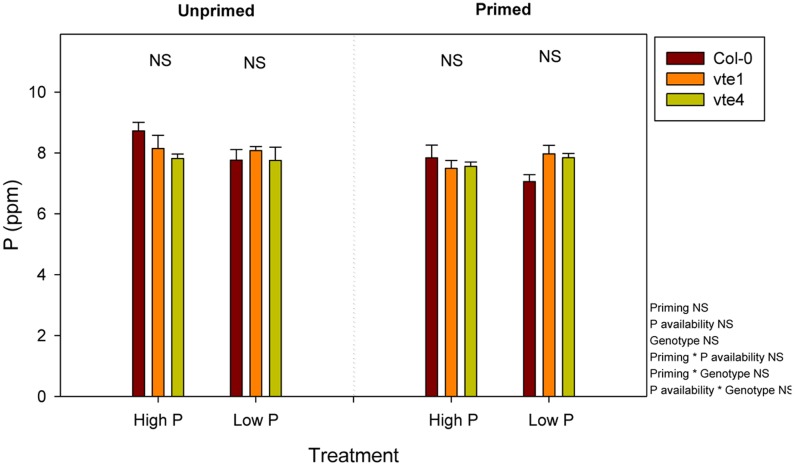
Endogenous P contents in vitamin E-deficient (*vte1* and *vte4* mutants) and wild-type plants of *A. thaliana* exposed to contrasting Pi availability, including unprimed and primed plants. Data represent the mean ± SE of *n* = 6 individuals. Significant differences between groups were tested by three-way analysis of variance (ANOVA, *P* < 0.05). NS, not significant. Results are expressed as parts per million (ppm) on a dry matter basis.

Rosette biomass was not affected by Pi availability, but it was significantly influenced by the genotype and priming. Plant biomass did not differ between genotypes under high Pi, either in unprimed or primed plants. However, rosette biomass was lower in the *vte1* mutant compared to wild-type plants at low Pi in unprimed plants, and in both mutants relative to the wild type in primed plants (**Figure 3**). Wild-type plants were the ones better adapted to low Pi in terms of biomass accumulation, so that priming had positive effects on plant growth. α-Tocopherol deficiency prevented the mutants to benefit from low Pi availability, as indicated by the smaller rosette biomass in both *vte* mutants compared to wild-type plants under primed conditions at low Pi (**Figure 3**). Despite these effects on growth, endogenous Pi concentrations did not differ between genotypes at any tested conditions (**Figure 4**). Furthermore, none of the other nutrients measured revealed any significant genotype-related difference (*P* < 0.05, ANOVA, Supplementary Figures 1, 2).

**FIGURE 5:**
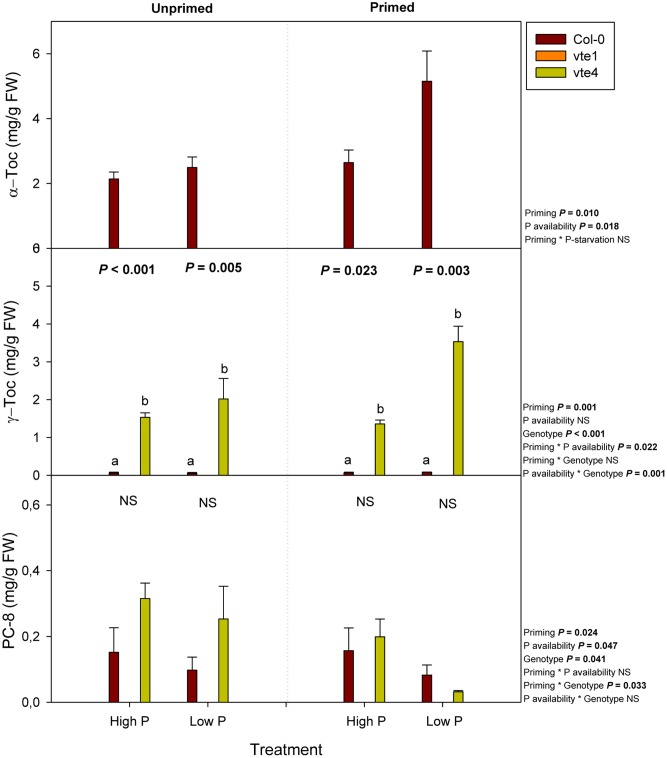
Endogenous contents of α-tocopherol, γ-tocopherol, and PC-8 in vitamin E-deficient (*vte1* and *vte4* mutants) and wild-type plants of *A. thaliana* exposed to contrasting Pi availability, including unprimed and primed plants. Data represent the mean ± SE of *n* = 6 individuals. Significant differences between groups were tested by three-way analysis of variance (ANOVA, *P* < 0.05). Different letters significant differences between genotypes at any given treatment (Duncan *post hoc* tests, *P* < 0.05). NS, not significant.

Tocochromanols, including α- and γ-tocopherol, as well as plastochromanol-8, were not detected in the *vte1* mutant, while both γ-tocopherol and plastochromanol-8 accumulated in the absence of α-tocopherol in the *vte4* mutant (**Figure 5**). In contrast, wild-type plants accumulated α-tocopherol in leaves, particularly at low Pi in primed condition. The contents of this antioxidant doubled at low Pi availability in primed compared to unprimed plants (**Figure 5**). The contents of γ-tocopherol also increased to a similar extent in the *vte4* mutant at low Pi in primed plants only. Plastochromanol-8 contents did not follow the same variations (**Figure 5**).

**FIGURE 6:**
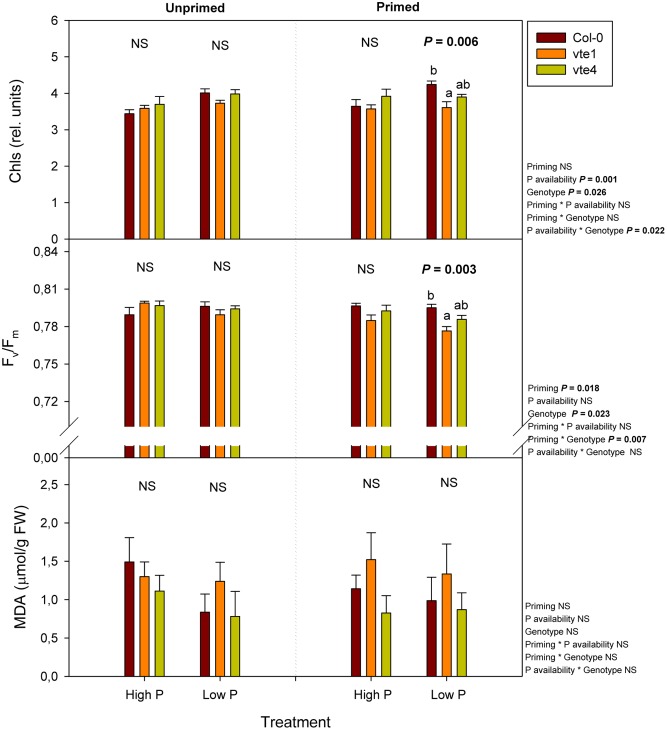
Total chlorophyll contents (given as relative units), maximum efficiency of PSII photochemistry (*F*_v_/*F*_m_ ratio), and malondialdehyde (MDA) accumulation, an indicator of the extent of lipid peroxidation in leaves, in vitamin E-deficient (*vte1* and *vte4* mutants) and wild-type plants of *A. thaliana* exposed to contrasting Pi availability, including unprimed and primed plants. Data represent the mean ± SE of *n* = 6 individuals. Significant differences between groups were tested by three-way analysis of variance (ANOVA, *P* < 0.05). Different letters significant differences between genotypes at any given treatment (Duncan *post hoc* tests, *P* < 0.05). NS, not significant.

Changes in photo-oxidative stress markers, including chlorophyll contents, the maximum efficiency of PSII photochemistry (*F*_v_/*F*_m_ ratio), and the extent of lipid peroxidation, estimated as MDA accumulation (**Figure 6**) paralleled those of rosette biomass (**Figure 3**), though effects were observed to a much more limited extent, particularly at low Pi in primed plants. Both chlorophyll contents and the *F*_v_/*F*_m_ ratio were lower in the *vte1* mutant compared to wild-type plants under primed condition at low Pi. However, reductions in the *F*_v_/*F*_m_ ratio were very small, the values in all plant genotypes being always above 0.75 (**Figure 6**). No significant differences in the extent of lipid peroxidation were observed between genotypes, Pi availability or priming (**Figure 6**).

### Differential Expression Pattern of TFs to Varying Pi Availability

Transcription factors, the major regulators of gene expression changes play a pivotal role in plant stress responses. To identify such transcriptional regulators that may have possible roles in the regulation of plant response to low Pi conditions and capture the priming effect on plant’s response to contrasting Pi availability, 1,880 Arabidopsis TFs expression was profiled under experimental conditions described above using quantitative real-time PCR (qRT-PCR). Obtained data was analyzed to identify TFs that respond differentially to varying low Pi conditions: (A) TFs responding to low Pi pre-treatment at high Pi, (B) TFs responding to a single low Pi episode, (C) Priming responsive TFs, and (D) TFs responding to recurrent low Pi stimuli (**Figure 2**).

**FIGURE 7:**
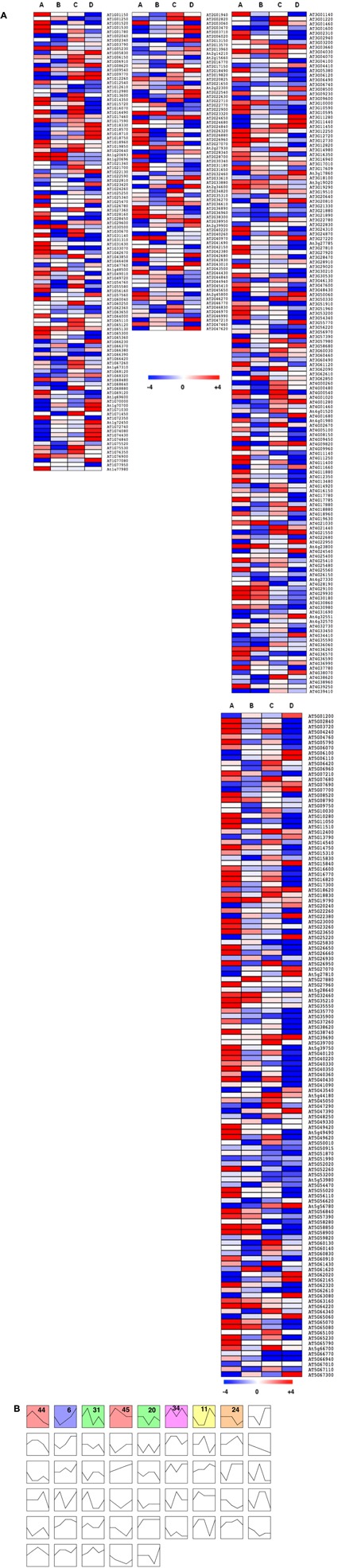
Changes in Pi availability trigger massive alterations in transcription factors (TFs) gene expression. **(A)** Heatmap showing the expression pattern of differentially expressed TFs (DETFs) under varying Pi availability in the wild type (groups A–D, respectively, from left to right). **(B)** Clustering analysis of DETFs using Short Time-series Expression Miner (STEM) software resulted in eight distinct cluster profiles that were significant among the 50 possible clusters obtained. For each genotype and treatment, data was obtained from six individuals, using the mean of two independent measurements from three pooled plants each.

In wild-type plants, a total of 454 TFs were differentially expressed with a 3-log_2_ fold change as cut-off (either up- or down-regulated) in any of the four comparisons performed, representing ∼ 24% of TFs tested in this study (**Figure 7A** and Supplementary Table 1). Diverse expression patterns of these TFs under different conditions tested suggest a massive transcriptional re-programming involved in plant response to Pi availability. Differentially expressed TFs (DETFs) represented several TF families such as MYB, AP2-EREBP, bZIP, bHLH, AGL, and BBX, among others. These TFs were manually classified based on their expression pattern as either A, B or C specific (Supplementary Table 2). The “A” group contains 69 TFs that were specifically up- (43) or down-regulated (26) in response to low Pi pre-treatment at high Pi, representing ∼15% of total DETFs. Forty-six TFs (∼10% of total DETFs) expressed specifically to a single low Pi episode (B), but only four of them were up-regulated. Interestingly, ∼12% of DETFs (29 up- and 24 down-regulated) were specifically expressed in response to priming treatment (C). These include several TFs functioning in response to plant growth or stress responses. For example, *SWI2C*, a core component of the SWI/SNF-type chromatin-remodeling complex C (CRCs), was up-regulated in a priming specific manner. SWI2C is a growth regulator and has been identified to interact with DELLA proteins (Sarnowska et al., 2013). Anthocyanin accumulation is a typical phenotypic response under Pi starvation (Morcuende et al., 2007); interestingly MYB111 and MYB113 involved in the regulation of anthocyanin production (Tohge et al., 2013) were repressed specifically in primed plants. Next, GO enrichment analysis was performed for the DETFs that showed priming specific expression using PLAZA 3.0 (Proost et al., 2015). Significantly over-represented GO terms describing the biological process include “gibberellin biosynthesis process,” “ABA-activated signaling pathway,” “regulation of triglyceride catabolic process,” and “histone H3- and H4-acetylation” (Supplementary Figure 3). These GO terms indicate the underlying mechanism of priming effect on plant response to low Pi (Supplementary Figures 4–6).

Additionally, in order to visualize cluster profiles of the DETFs based on their expression direction and magnitude, STEM (STEM) was employed. A comparison of observed groups with those expected in random permutation enables to determine enrichment of the obtained clusters (Ernst et al., 2005; Ernst and Bar-Joseph, 2006). Such comparison of expression patterns resulted in profiles classified into 50 categories in the response of wild-type plants to contrasting Pi availability (**Figure 7B**). Out of the 50 possible clusters, eight clusters were found to be significant (*P*-value ≤ 0.05). Colored blocks in (**Figure 7B** and Supplementary Table 3) display expression profiles of significant clusters (clusters 6, 11, 20, 24, 31, 34, 44, and 45) arranged based on their significance. MYB TF family represented the highest number among the various cluster profiles, followed by AGL and bHLH TF families. Cluster profiles 6, 44, and 31 displayed 73, 67, and 47 TFs, respectively, representing larger cluster groups, among others. To understand the biological significance of the obtained cluster profiles, GO terms enriched in the TF clusters were analyzed. Profile 6 is enriched for TFs associated with GO terms “regulation of response to stimulus,” “response to jasmonic acid, endogenous stimulus, hormone, stress, acid chemical, oxygen containing compound, gibberellin, salicylic acid, and ethylene,” “regulation of signal transduction, jasmonic acid-mediated signaling pathway,” and “chromatin modification and organization,” among others. Profile 44 represents GO terms “regulation of cellular macromolecule biosynthetic process,” “nucleobase-containing compound metabolic process,” “organic substance biosynthesis and metabolic process,” and “root system development,” among others. Profile 31 represents “response to salt and osmotic stress, alcohol, lipid.”

### Vitamin E Deficiency Alters Plant Response to Contrasting Pi Availability

**FIGURE 8:**
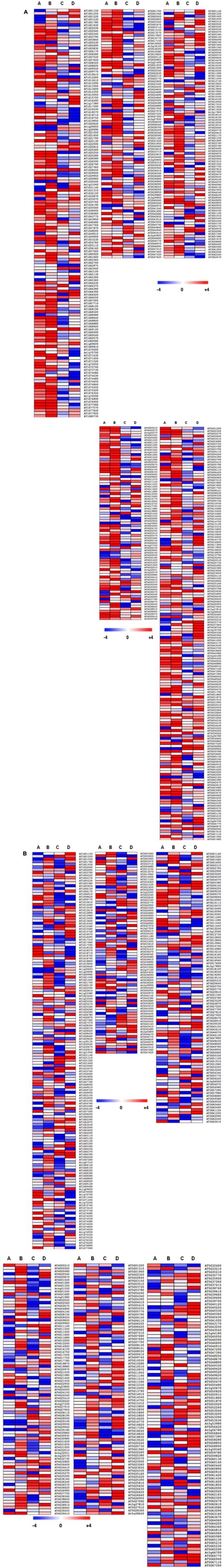
Effect of vitamin E deficiency on TFs profile under contrasting Pi availability. Heatmap showing the expression pattern of DETFs under varying Pi conditions in *vte1*/wild type **(A)** and *vte4*/wild type **(B)**. Results for comparisons A–D, respectively, are shown from left to right. For each genotype and treatment, data was obtained from six individuals, using the mean of two independent measurements from three pooled plants each.

Given the extensive expression changes of TFs under varying Pi availability in wild-type plants (**Figure 7A**), it was interesting to study how an altered tocopherol composition affects the expression of those transcriptional regulators under contrasting Pi availability. Toward this, we performed whole TF profiling in *vte1* and *vte4* mutants under the above described conditions (**Figure 2**). A total of 568 and 583 TFs were differentially expressed (both up- and down-regulated with 3-log_2_ fold change as cut-off threshold) in *vte1* and *vte4* mutants compared to the wild type (**Figure 8** and Supplementary Tables 4, 5) representing ∼30 and ∼31% of the total TFs analyzed, respectively. DETFs in the *vte1* mutant are represented by a mixture of TF families, while TFs belonging to MYB, AGL, bZIP, bHLH TF families were abundant in the *vte4* mutant compared to the wild type. To identify TFs that exhibit condition specific expression patterns, manual classification was performed (Supplementary Tables 6, 7) as described above (see also **Figure 2**). In the *vte1* mutant, 49 TFs were specific to group A of which, 34 and 15 were up- and down-regulated, respectively, representing ∼9% of the total DETFs. *vte4* mutant displayed ∼8% TFs specific to group A with 14 and 29 up- and down-regulated, respectively. Contrasting to the wild type, the *vte1* mutant displayed more TFs specific to group B with 121 up-regulated and only 1 down-regulated (∼22% of DETFs). In the *vte4* mutant, around 8% (35 up- and 8 down-regulated) of total DETFs responded specifically to group B. The TFs involved in the regulation of anthocyanin were up-regulated in both the mutants compared to wild-type plants, suggesting that the mutants were experiencing stress under low Pi compared to the wild type. For example, in the *vte1* mutant, *MYB112* was induced in group A and *MYB114* up-regulated in both A and B, both known to be involved in the regulation of anthocyanin production (Tohge et al., 2013; Lotkowska et al., 2015). Furthermore, ∼8% TFs displayed a priming specific (group C) expression pattern, of which 28 and 16 were up- and down-regulated in the *vte1* mutant compared to the wild type. Interestingly, several of these TFs showed an opposite priming specific expression pattern in wild-type plants. Priming repressed TFs in the wild type, *MYB111* and *MYB113* expression was induced in *vte1* primed plants, which suggests that these plants could not benefit from priming.

Varying number of TFs representing group specific expression pattern among *vte1* and *vte4* mutants was observed which might suggest diverse roles for the different tocopherol forms. As contrasting to *vte1*, *vte4* mutants displayed ∼24% (56 up- and 81 down-regulated) TFs with priming specific expression pattern compared to the wild type. *MYB111* expression was also increased in primed plants. Expression of *ZAT6*, a negative regulator of Pi homeostasis (Devaiah et al., 2007b) was up-regulated in the *vte4* mutant in a priming specific manner. Previously, expression of several ethylene signaling pathway genes such as *EIN2*, *EIN3*, *CTR1*, and *ERF1* has been reported to be up-regulated in the *vte4* mutant upon salt stress compared to wild-type plants (Cela et al., 2011). In the present study, expression of several ERFs was induced, while *EIN* and *EIL3* expression was repressed in primed *vte4* plants. GO enrichment analysis of the priming specifically expressed TFs revealed interesting over-represented GO terms such as “ethylene mediated signaling pathway,” “cytokinin activated signaling pathway,” “TF import to nucleus,” and “histone H3 K27 methylation” in *vte1* mutant; “salicylic acid-mediated signaling pathway,” “sucrose-induced translational repression,” and “regulation of pectin biosynthesis and metabolism” in *vte4* mutant. Activation of cytokinin signaling pathway in *vte1* mutant indicate attenuated low Pi response in these plants, as cytokinins negatively regulate Pi starvation responses (Yang and Finnegan, 2010). Defense hormone SA-mediated signaling and pectin biosynthesis in the *vte4* mutant suggests activated defense response networks in *vte* mutants in response to priming. Further, positive effect of priming on the growth observed in the wild type was compromised in *vte* mutants possibly due to the tradeoff toward defense.

**FIGURE 9:**
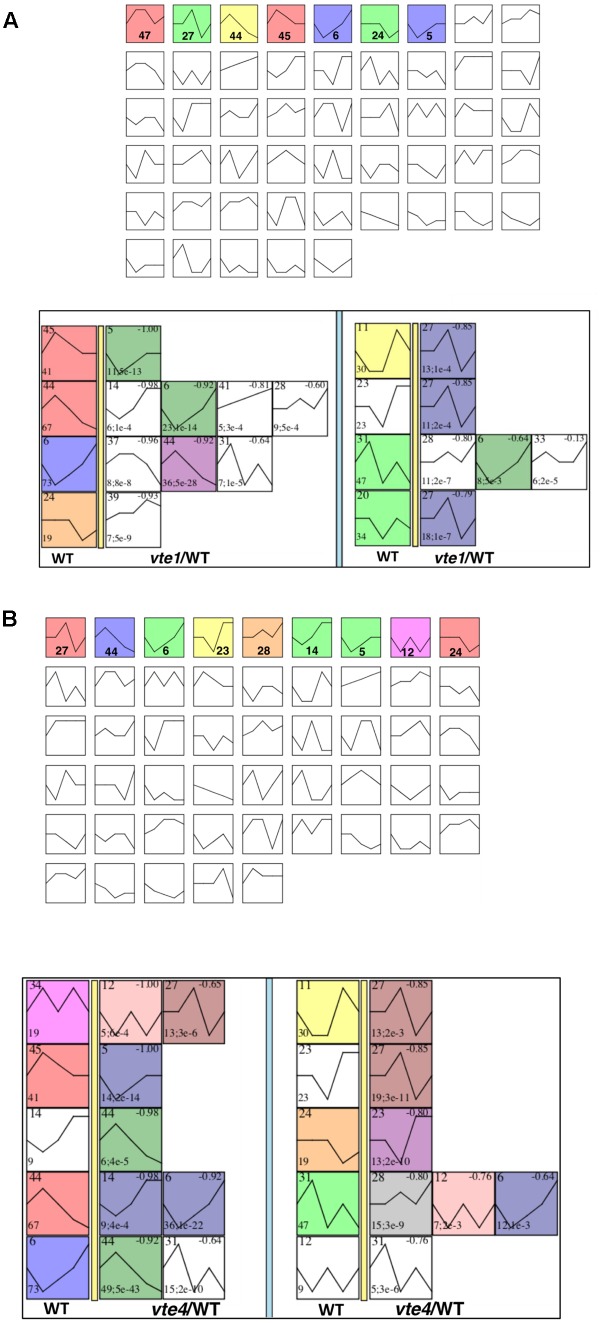
Vitamin E deficiency alters the expression pattern of TFs to contrasting Pi availability. Cluster profiles of DETFs obtained using STEM software, significant profiles are represented in colored blocks (upper panel), blocks displaying the comparison of wild type with *vte* mutant profiles (lower panel). **(A)** *vte1*/wild type and **(B)** *vte4*/wild type. For each genotype and treatment, data was obtained from six individuals, using the mean of two independent measurements from three pooled plants each.

Next, to understand those expression patterns observed among different Pi availability conditions in both the mutants, first we clustered the DETFs into profiles using STEM. A total of 50 possible cluster profiles were obtained for both mutants, of which, 7 (5, 6, 24, 27, 44, 45, and 47) and 9 (5, 6, 12, 14, 23, 24, 27, 28, and 44) clusters were found to be significant in *vte1* and *vte4* mutants, respectively (**Figures 9A,B** and Supplementary Tables 8, 9). Further, these significant clusters were compared with the significant clusters obtained from wild-type plants using STEM, where the TFs in each cluster from the mutants were compared with their magnitude of expression and direction in the wild type. Interestingly, such correlation revealed opposite patterns for many of the cluster profiles between the mutants and the wild type (**Figures 9A,B**). To learn about the involvement of tocopherols in mediating the plant response to contrasting Pi availability, these oppositely expressed TFs could serve as a valuable resource. Additionally, learning about the GO terms associated with these TFs would help to expand our knowledge in understanding the adaptive mechanisms associated with vitamin E under varying Pi availability. Interestingly, the largest cluster profiles 6 and 44 of wild-type plants showed good correlation with the cluster profiles of the *vte1* mutant. Wild type profile 6 and 44 in the *vte1* mutant displayed TFs representing similar GO terms such as “response to: gibberellin, hormone, endogenous stimulus, organic substance, ethylene, lipid, jasmonic acid, salicylic acid, auxin, ABA, organic cyclic compound,” “regulation of: signaling, signal transduction, cell communication,” and “negative regulation of: cellular, macromolecule biosynthesis process, nucleobase-containing compound metabolic process” among others. Wild type profile 44 correlated with *vte1* profile 6 representing the GO terms “heterocyclic compound-, organic cyclic compound-, nucleic acid-binding,” and “protein dimerization activity.” Profile correlations between wild type and the *vte4* mutant as well showed significant GO enrichment terms. Wild type profile 6 and *vte4* profile 44 correlated TFs represented GO terms “response to gibberellin, stress, abiotic stress, osmotic stress, lipid, salicylates, ethylene,” “chromatin modification, organization,” and “histone modification,” among others. Wild type profile 23 and *vte4* profile 24 displayed GO terms “cellular response to: ethylene stimulus, hormone stimulus, organic substance, endogenous and chemical stimulus,” “ethylene activated signaling pathway,”, “phosphorelay signal transduction system,” and “hormone-mediated signaling pathway”.

### Hormonal Profiling Reveals Activated Defense Response in *vte* Mutants under Contrasting Pi Availability

**FIGURE 10:**
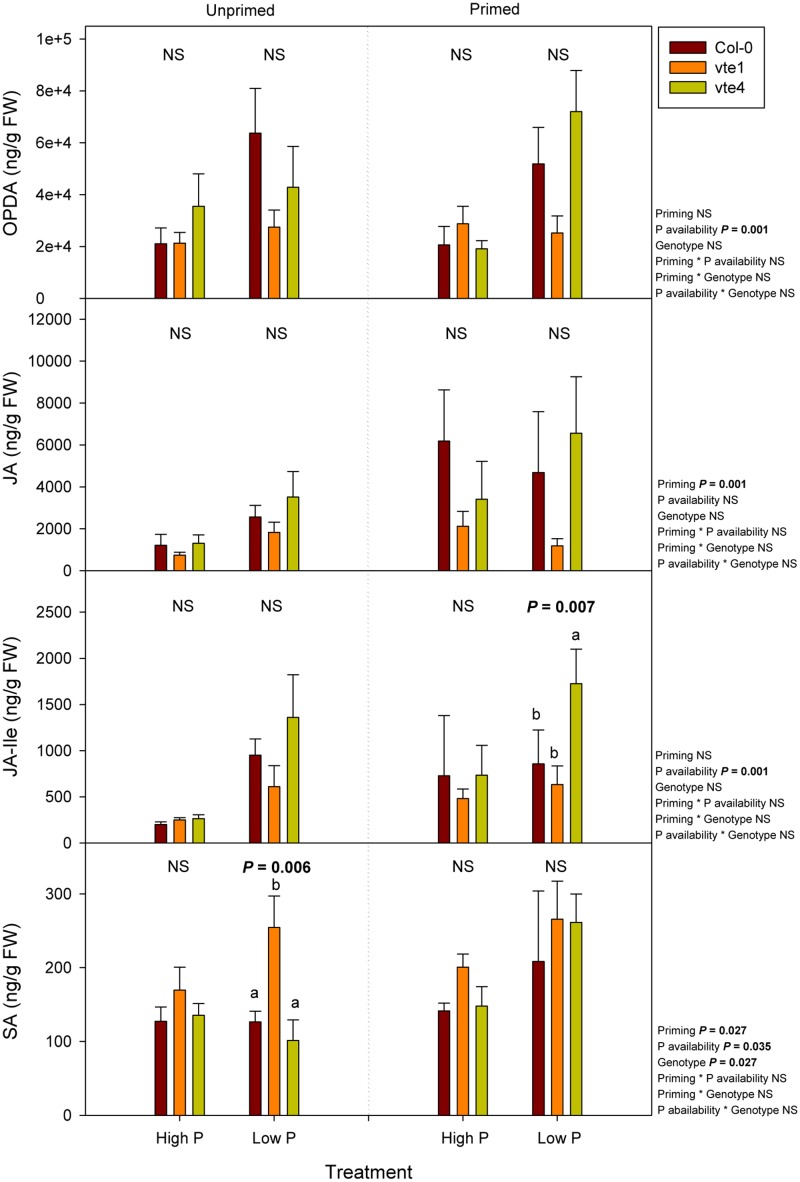
Endogenous concentrations of jasmonates, including *oxo*-phytodienoic acid (OPDA), jasmonic acid (JA) and jasmonic acid isoleucine (JA-Ile), and salicylic acid (SA) in vitamin E-deficient (*vte1* and *vte4* mutants) and wild-type plants of *A. thaliana* exposed to contrasting Pi availability, including unprimed and primed plants. Data represent the mean ± SE of *n* = 6 individuals. Significant differences between groups were tested by three-way analysis of variance (ANOVA, *P* < 0.05). Different letters significant differences between genotypes at any given treatment (Duncan *post hoc* tests, *P* < 0.05). NS, not significant.

Hormonal profiling revealed genotype-related differences, particularly for salicylic acid contents, which increased significantly in the *vte1* mutant compared to wild-type plants at low Pi, but in unprimed plants only. This effect was not observed in primed plants, because in this case salicylic acid contents increased similarly in the three genotypes at low Pi availability (**Figure 10**). Furthermore, enhanced jasmonic acid-isoleucine (JA-Ile) contents were observed in the *vte4* mutant compared to wild-type plants and the *vte1* mutant in primed plants at low Pi (**Figure 10**). No genotype-related differences were observed in the contents of cytokinins (Supplementary Figure 3), auxin (Supplementary Figure 7), gibberellins (Supplementary Figure 8), ABA, the ethylene precursor, 1-amino-cyclopropane-1-carboxylic acid, or melatonin (Supplementary Figure 9). Priming had significant effects on jasmonates, salicylates, and auxin contents; defense-related compounds such as jasmonic acid in particular, increasing, and indole-3-acetic acid contents decreasing, in primed plants (**Figure 10** and Supplementary Figure 7). Low Pi availability increased the contents of *oxo*-phytodienoic acid and JA-Ile irrespective of priming, the latter particularly increasing in the *vte4* mutant in primed plants (**Figure 10**).

## Discussion

Phosphorus is one of the crucial macronutrients needed for the plants and its limitation leads to adaptations both at molecular, biochemical, and developmental level (Marschner, 2012). Our current study has shown that repeated exposure (priming) of plants to moderately low Pi availability condition improves growth in the model plant *A. thaliana*. Priming had a positive effect on plant growth in the wild type, but this effect was abolished in both *vte* mutants. Priming led to significant increases in α-tocopherol contents in the wild type, thus indicating α-tocopherol deficiency in both the *vte1* and *vte4* mutants may explain the genotype-related effects observed in the present study. It is noteworthy that both wild-type plants and the *vte4* mutant increased tocopherol levels (α- and γ-tocopherol, respectively) instead of those of plastochromanol-8 in response to priming, thus genotype-related effects on growth in primed plants may be related to changes in tocopherols rather than plastochromanol-8. Despite platochromanol-8 antioxidant role in thylakoid membranes (Kruk et al., 2014), it seems that tocopherols play a prominent role over plastochromanol-8 in regulating plant response to contrasting Pi availability in *A. thaliana*. Vitamin E deficiency had slight effects on photoinhibition (as indicated by reductions in chlorophyll levels and the *F*_v_/*F*_m_ ratio), particularly in the *vte1* mutant, but differences between genotypes were very small and lipid peroxidation (as indicated by MDA accumulation) kept unaltered, thus indicating that genotype-related effects on growth in primed plants might be mostly associated with mechanisms other than a slightly enhanced photo-oxidative stress in leaves due to α-tocopherol deficiency. Here, it is shown that an alteration in the vitamin E composition and contents in chloroplasts may influence growth and defense though modulation of specific clusters of gene expression and hormones.

Transcription factors regulate the majority of gene expression changes and thus play a crucial role in regulating the plant response to various stresses including Pi limitation (Wu et al., 2003). Several studies have focused on identifying such TFs that are involved in the regulation of plant response to Pi limitation. For example, Arabidopsis MYB62, ZAT6, bHLH32, PTF1, WRKY75 and rice OsWRKY74 are of those TFs whose role in the regulation of Pi response have been identified (Yi et al., 2005; Chen et al., 2007; Devaiah et al., 2007a,b, 2009; Dai et al., 2016). Previously, genome wide expression profiling revealed specific sets of TFs to be involved in regulation of early and late Pi deficiency responses (Misson et al., 2005). In this study, qRT-PCR analyses of 1,880 TF genes revealed massive transcriptional reprogramming in response to different phosphate regimes. Our data show that priming had a clear positive effect on the response to Pi limitation in wild-type plants. Transcript levels of 45 TFs were specifically deregulated (29 up- and 24 down-regulated) in response to priming treatment. These TFs are mainly associated with regulation of plant growth or response to stresses. Moreover, among TFs specifically repressed in response to priming, *MYB111* and *MYB113*, TFs involved in anthocyanin biosynthesis, were identified. This observation suggests that pre-exposure to a moderate Pi limitation renders the plant to efficiently safeguard when encountered with a second stress. Furthermore, priming specific induction of TF *SWI2C* involved in cross-talking with several hormonal pathways indicate a possible involvement of hormones in regulating the priming specific responses. Interestingly, priming specific TFs represent sets of TFs involved in stress responses such as *MYB102*, *ZAT12*, *MYB4R1*, *WOXY9A*, *HB22*, *HB52*, and *ANAC047*; disease responsive like *WRKY16* and *HAT3.1*; hormone related such as *AtABF1*, *ABI4*, *ABI5*, *ETHYLENE INSENSITIVE 3 FAMILY PROTEIN*, and *ERF13*; development related like *SPL7*, *SPL12*, and *ULTULT1*; and *NLP5*, a TF involved in nitrate signaling. Enriched GO terms for the priming specifically expressed TFs display several interconnected pathways involved in the regulation of plant growth and stress responses. Regulation of growth promoting hormone (gibberellin) biosynthesis correlated well with the increased rosette biomass of the wild-type plants under primed condition. Previous studies reported ABA to have a minimal role in mediating low Pi responses (Franco-Zorrilla et al., 2004), whereas in the current study, enriched ABA-activated signaling in primed plants may suggest its possible role in regulating efficient stress response. Post-translational modifications of histones at specific amino acid residues such as acetylation, SUMOylation (Small ubiquitin-related modifier), ubiquitination, phosphorylation indicates the integrity of the nucleosome in that region (Berger, 2007). In Arabidopsis Pi deficiency response pathway, At-SIZ1 was identified to function as a SUMO E3 ligase, which can mediate SUMOylation of AtPHR1. It can also associate with a putative ubiquitin conjugase AtPHO1/UBC24 in the SUMOlylation pathway (Yang and Finnegan, 2010). Repression of target genes by endogenous or environmental cues can be achieved through reduction in histone acetylation levels, thus acetylation of histones is associated with gene activation (Dhar et al., 2014). Interestingly, histone H3 and H4 acetylation was over-represented in GO enrichment analysis for the priming specific TFs. It would be interesting to understand the possible link between histone acetylation and Pi responses, in particular upon priming.

Both *vte* mutants displayed distinct TF expression profile compared to wild-type plants under moderately low Pi availability. Induction of *MYB112* and *MYB114* (TFs involved in anthocyanin production) in the *vte1* mutant, and induction of *MYB62* (a repressor of Pi homeostasis) in both *vte1* and *vte4* mutants compared to the wild type indicate attenuated responses to Pi limitation in these plants. Furthermore, the *vte1* mutant displayed opposite expression pattern for several of the priming specific TFs found in wild-type plants, which may be linked to the lack of α-tocopherol in these mutants. Functional characterization of these TFs might give more insights into the role of α-tocopherol in regulating the plant response to Pi availability. Massive priming specific TF expression changes were also observed in the *vte4* mutant upon varying Pi availability, which include several TF families such as WRKY, bZIP, GATA, NAC, and ERF. GO enrichment analysis clearly indicates the potential role of tocopherols in regulating priming. Activated cytokinin-mediated signaling pathway marks the underlying attenuated low Pi response in *vte1* mutant. More interestingly, *vte1* mutants display heterochromatin state; as methylation of H3 lysine, especially H3K27me3 has been identified to be a major chromatin silencing modification associated with 1000s of genes at the 5′ region (Zhang et al., 2007; Dhar et al., 2014). Activated TF import into nucleus in the *vte1* mutant provides a mechanism to translate signals from the cytosol to the nucleus, thus indicating vitamin E deficiency may profoundly alter signaling processes. Activated defense responses in *vte4* mutants suggest a possible tradeoff regulation between growth and defense response. Furthermore, sucrose-induced translational repression in the *vte4* mutant indicates sugar signaling is strongly influenced by the tocopherol composition, an aspect that has also been shown in salt-stressed potato plants (Asensi-Fabado et al., 2015).

Further, comparing the cluster profiles of wild-type plants and the *vte* mutants displayed clusters enriched with opposite TF expression patterns. Interestingly, the GO terms associated with those clusters are “response to gibberellin, jasmonates, salicylates, ABA, ethylene, lipid, auxin, organic cyclic compound” and “negative regulation of cellular, macromolecular biosynthesis process,” among others. Changes related to growth promoting hormone gibberellin and stress hormones such as salicylates, jasmonates, ABA, and ethylene may further support a possible tradeoff scenario in these mutants compared to the wild type. Indeed, results of hormonal profiling confirms a tradeoff between response to contrasting Pi availability and activation of defense-related compounds with increases in endogenous salicylic acid concentrations in the *vte1* mutant compared to the wild type at low Pi in unprimed plants, and enhanced JA-Ile levels in the *vte4* mutant compared to the wild type at low Pi in primed plants. In these two cases, these mutants grew less than the wild type and activated more chemical defenses. It is therefore likely that reduced growth and photoprotection in vitamin E-deficient mutants favors the capacity to synthesize chemical defenses, such as salicylates and jasmonates, under abiotic stress conditions, thus suggesting a tradeoff between growth and different defense pathways in plants (growth and photoprotection versus potential chemical defense to biotrophs and necrotrophs through salicylates and jasmonates, respectively), which is in agreement with previous studies (Demmig-Adams et al., 2013, 2014; Morales et al., 2015; Simancas and Munné-Bosch, 2015).

The present study shows a link between the capacity of plants to synthesize chloroplastic antioxidants and massive changes in gene expression, therefore suggesting vitamin E influences retrograde signaling an aspect that has been previously proposed (Hofius et al., 2004; Sattler et al., 2004; Munné-Bosch, 2005; Cela et al., 2011; Pfannschmidt and Munné-Bosch, 2013). In this respect, it has been previously shown that tocopherols play a major role in the regulation of fatty acid metabolism, not only from chloroplasts, but also from the endoplasmic reticulum, due to a continuous exchange of information between endoplasmic reticulum and chloroplast membranes that may help transfer signals from chloroplasts to the nucleus (Sattler et al., 2006; Mehrshahi et al., 2013, 2014). In the present study, pre-treatment with moderately low Pi (condition A) led to down- and up-regulation of MYB30 in the wild type and the *vte4* mutant, respectively. MYB30 is a TF that regulates very-long-chain fatty acid biosynthesis (Raffaele et al., 2008), therefore suggesting a link between vitamin E and fatty acid metabolism in plant response to low Pi. Furthermore, alterations in fatty acid metabolism due to the effects of tocopherol deficiency (in the *vte1* mutant) or an altered tocopherol composition (in the *vte4* mutant) may lead to profound changes in lipid peroxidation products, including alterations in both enzymatic (as shown here with jasmonate levels in the *vte4* mutant) and non-enzymatic lipid peroxidation products, an aspect that warrants further investigation in *vte* mutants exposed to contrasting Pi availability. It is concluded that α-tocopherol may play a major role in plant response to contrasting Pi availability not only protecting plants from photo-oxidative stress, but also exerting a regulatory role on growth and defense though modulation of specific clusters of gene expression and hormones. Further research is, however, needed to better understand the metabolic and cellular processes linking vitamin E with retrograde signaling in plants.

## Author Contributions

SM-B and SB conceived the research plans. AA and BS performed the experiments. AA and SM-B wrote the article with contributions of BS and SB.

## Conflict of Interest Statement

The authors declare that the research was conducted in the absence of any commercial or financial relationships that could be construed as a potential conflict of interest.

## Footnotes

1 http://www.tm4.org/
