# Supplementary material for: Defense-Related Transcriptional Reprogramming in Vitamin E-Deficient Arabidopsis Mutants Exposed to Contrasting Phosphate Availability
Source: Front Plant Sci. 2017 Aug 10;8:1396. doi: 10.3389/fpls.2017.01396 (PMC5554346; doi:10.3389/fpls.2017.01396)

Suppl. Fig. 1. Endogenous S, Ca, Mg, K, Na and Mn levels in vitamin E-deficient (*vte1* and *vte4* mutants) and wild type plants of *Arabidopsis thaliana* exposed to contrasting Pi availability, including unprimed and primed plants. Data represent the mean ± SE of n=6 individuals. Significant differences between groups were tested by three-way analysis of variance (ANOVA, P<0.05). Different letters significant differences between genotypes at any given treatment (Duncan posthoc tests, *P*<0.05). NS, not significant. Results are expressed as parts per million (ppm) on a dry matter basis.

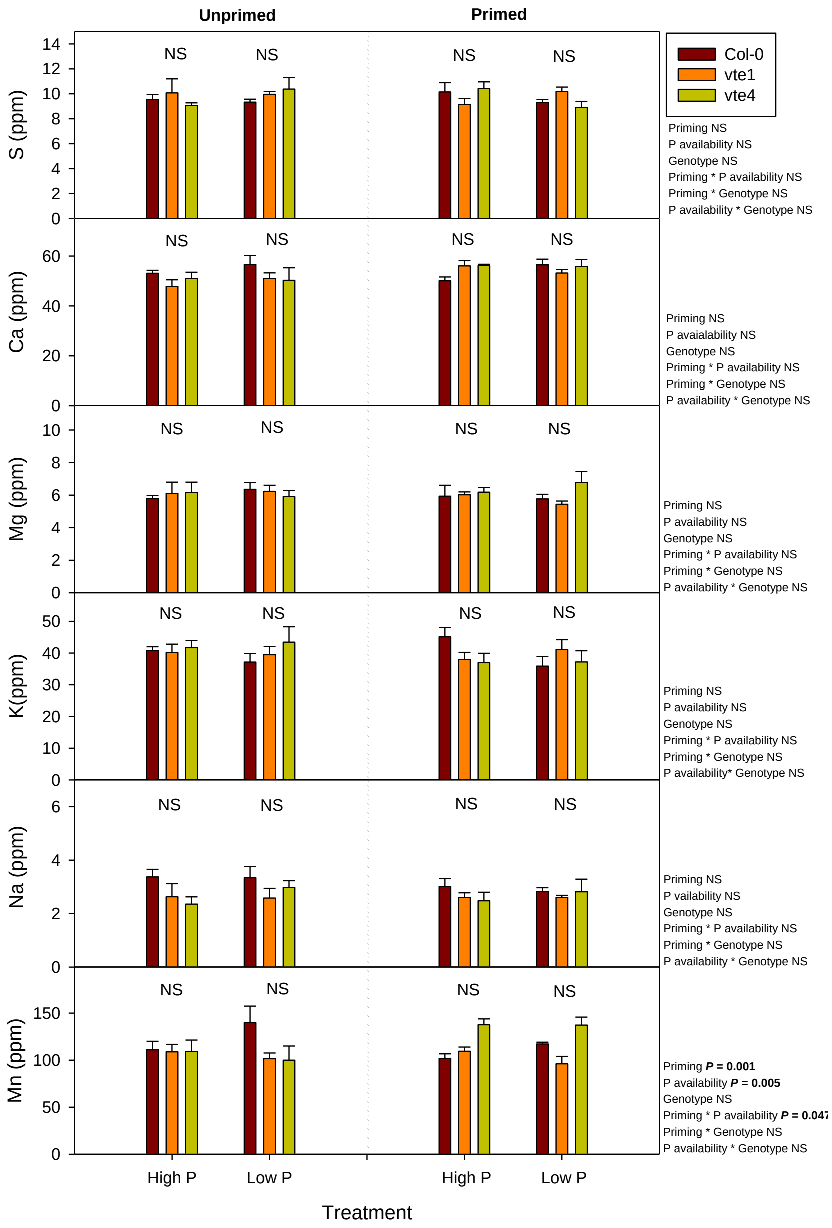

Suppl. Fig. 2. Endogenous Zn, Fe, Mo, B, Cu and Si levels in vitamin E-deficient (*vte1* and *vte4* mutants) and wild type plants of *Arabidopsis thaliana* exposed to contrasting Pi availability, including unprimed and primed plants. Data represent the mean ± SE of n=6 individuals. Significant differences between groups were tested by three-way analysis of variance (ANOVA, P<0.05). Different letters significant differences between genotypes at any given treatment (Duncan posthoc tests, *P*<0.05). NS, not significant. Results are expressed as parts per million (ppm) on a dry matter basis.

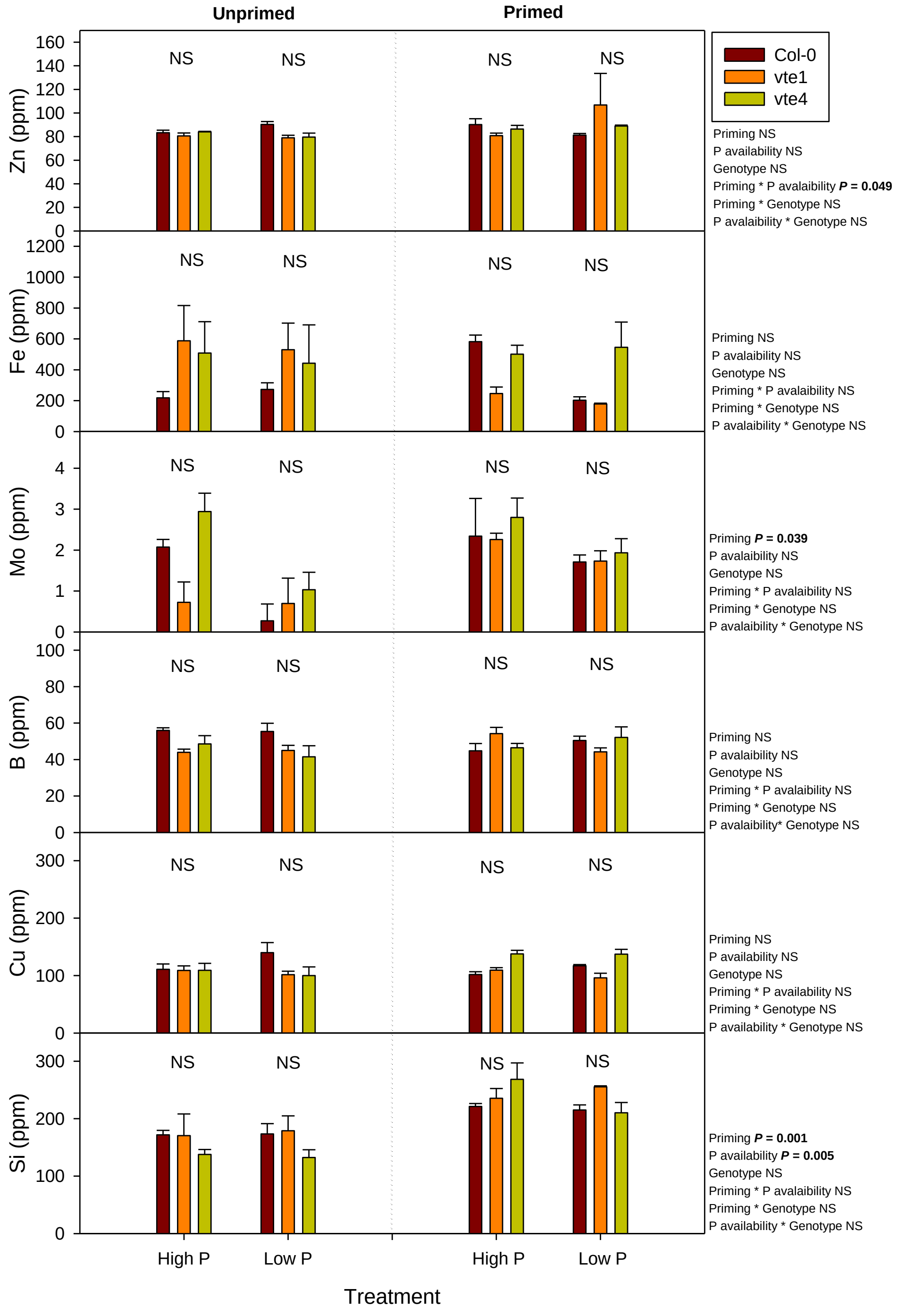

Suppl. Fig. 3. Endogenous concentrations of cytokinins, including *trans*-zeatin (*t*-Z), *trans*-zeatin riboside (*t*-ZR), isopentenyladenosine (IPA) and 2-isopentenyladenine (2iP) in vitamin E-deficient (*vte1* and *vte4* mutants) and wild type plants of *Arabidopsis thaliana* exposed to contrasting Pi availability, including unprimed and primed plants. Data represent the mean ± SE of n=6 individuals. Significant differences between groups were tested by three-way analysis of variance (ANOVA, *P*<0.05). Different letters significant differences between genotypes at any given treatment (Duncan posthoc tests, *P*<0.05). NS, not significant.

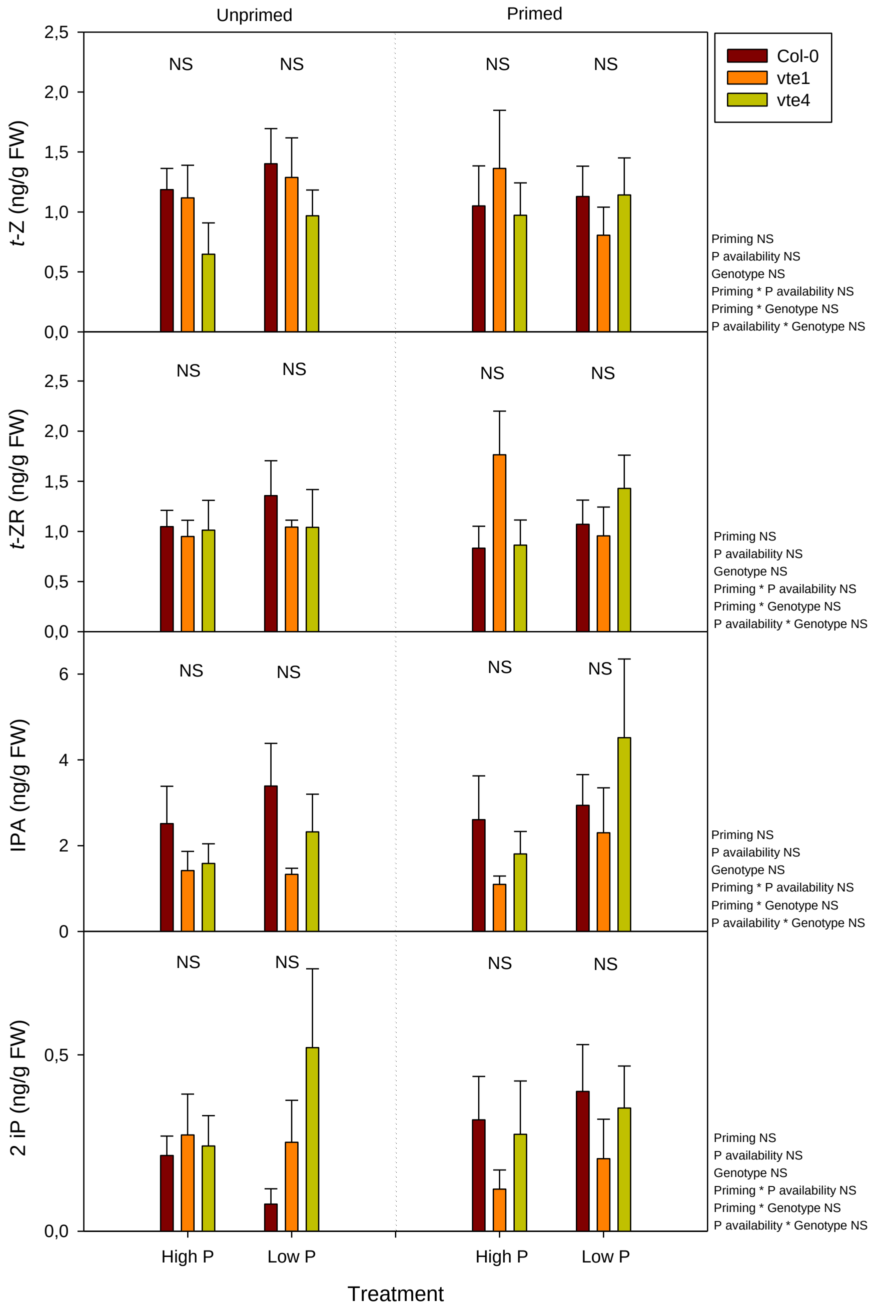

Suppl. Fig. 4. GO enrichment analysis for priming responsive TFs in wild-type plants. Several significantly over-represented GO terms (*P*<0.05) were obtained for the priming responsive differentially expressed TFs from wild type plants using GO enrichment analysis tool in PLAZA 3.0.

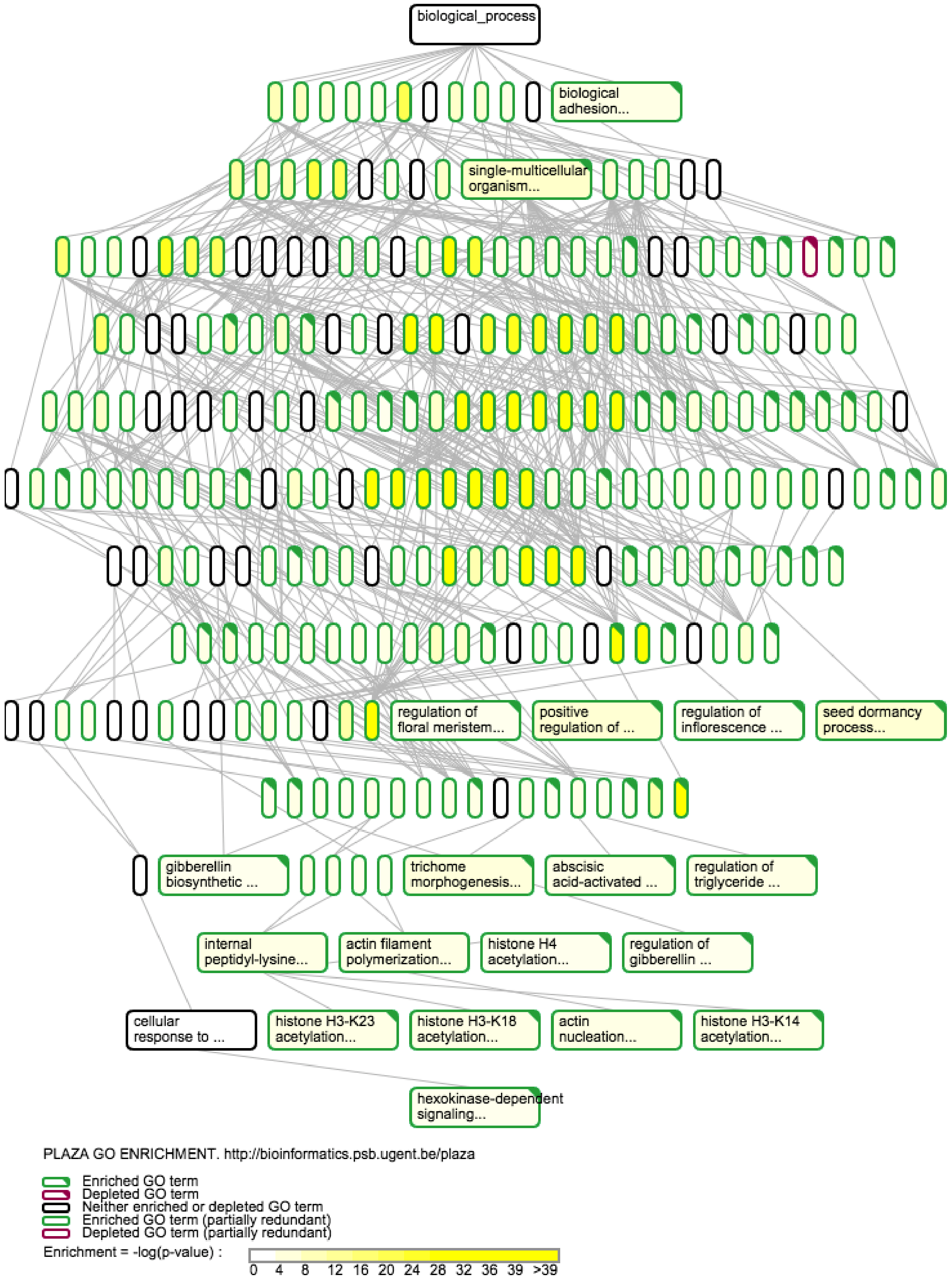

Suppl. Fig. 5. GO enrichment analysis for priming responsive TFs in *vte1*/wild type plants. Several significantly over-represented GO terms (P<0.05) were obtained for the priming responsive differentially expressed TFs from *vte1*/WT plants using GO enrichment analysis tool in PLAZA 3.0.

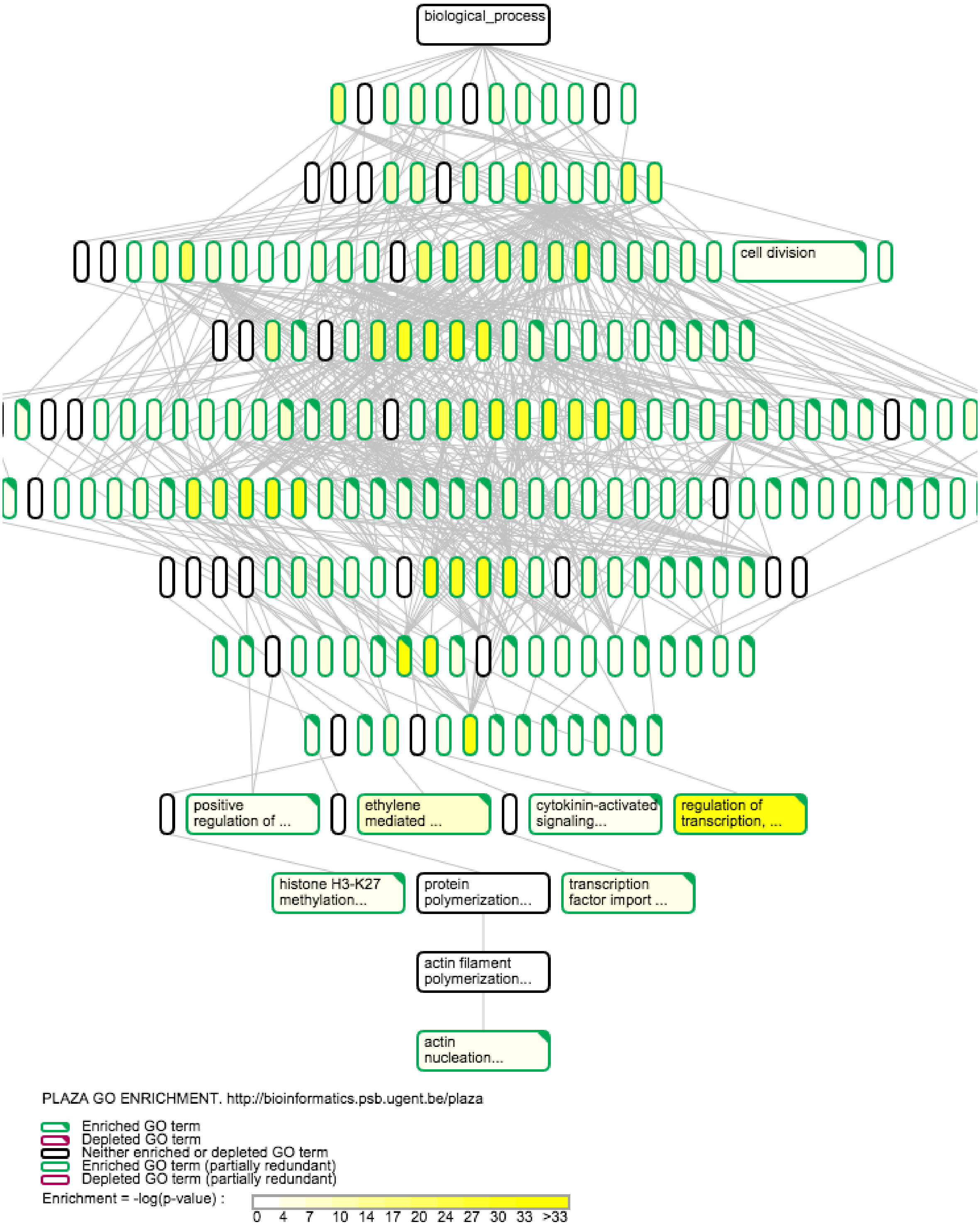

Suppl. Fig. 6. GO enrichment analysis for priming responsive TFs in *vte4*/wild type plants. Several significantly over-represented GO terms (P<0.05) were obtained for the priming responsive differentially expressed TFs from *vte4*/WT plants using GO enrichment analysis tool in PLAZA 3.0.

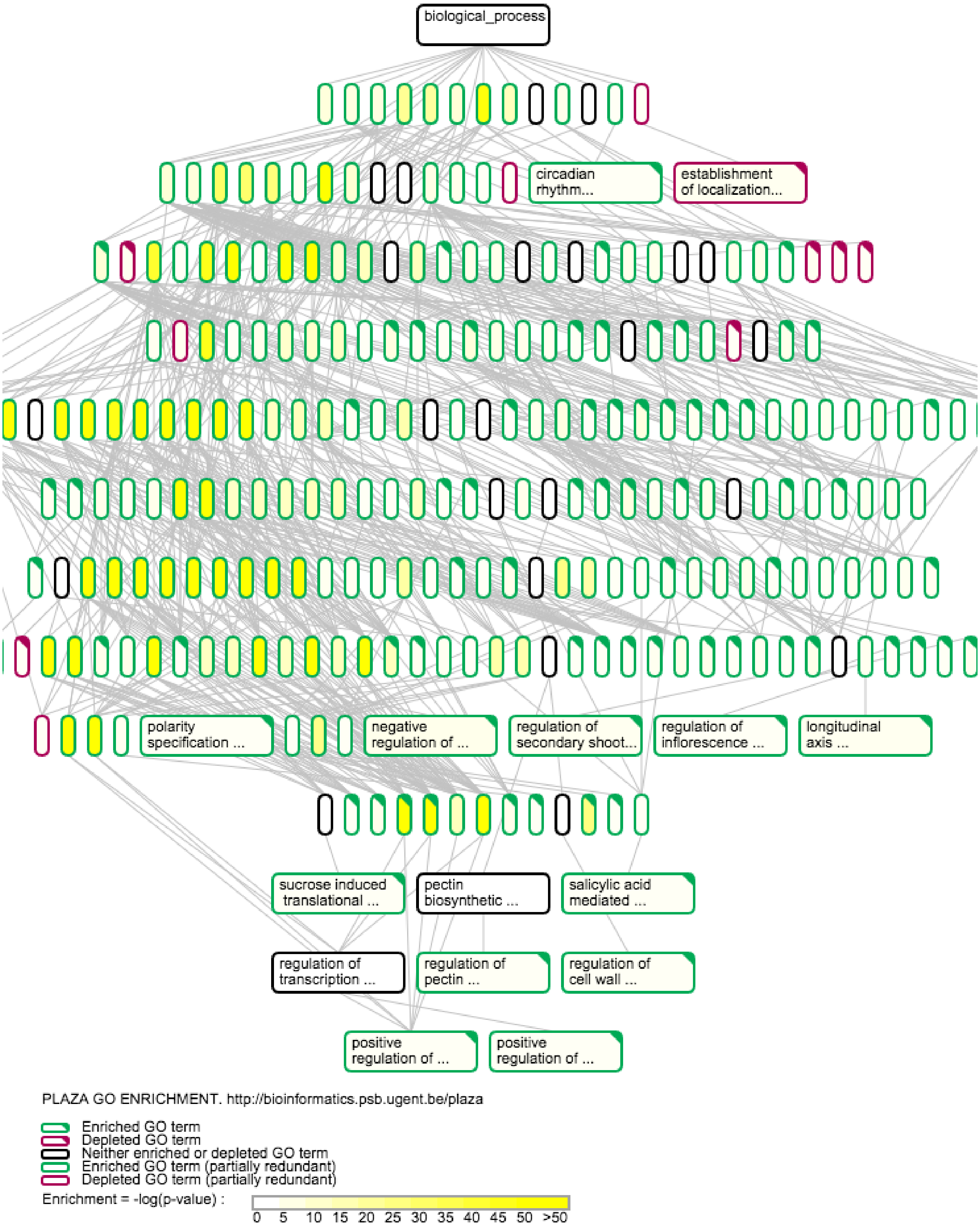

Suppl. Fig. 7. Endogenous concentrations of the auxin, indole-3-acetic acid (IAA) in vitamin E-deficient (*vte1* and *vte4* mutants) and wild type plants of *Arabidopsis thaliana* exposed to contrasting Pi availability, including unprimed and primed plants. Data represent the mean ± SE of n=6 individuals. Significant differences between groups were tested by three-way analysis of variance (ANOVA, *P*<0.05). Different letters significant differences between genotypes at any given treatment (Duncan posthoc tests, P<0.05). NS, not significant.

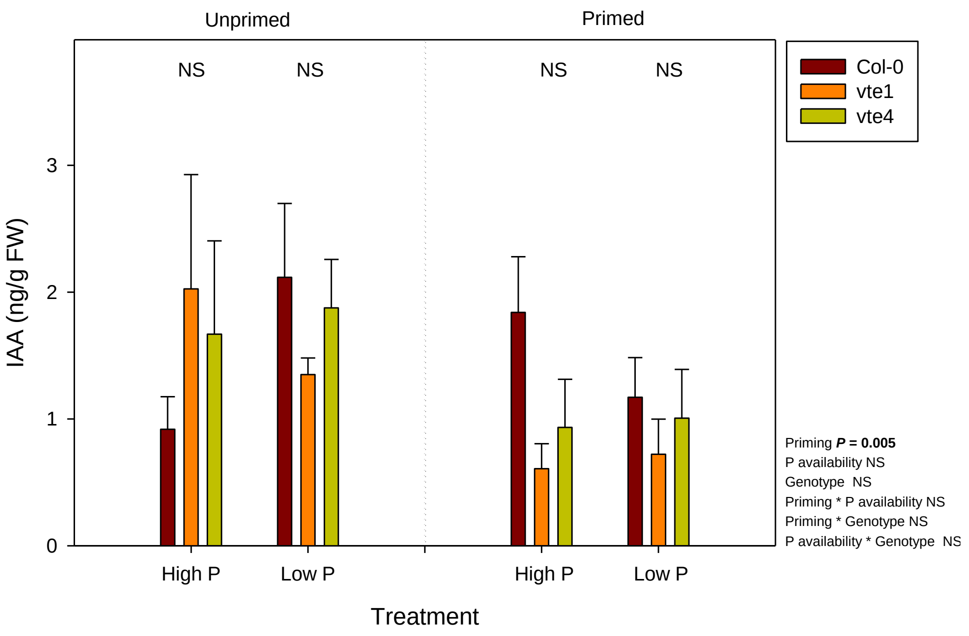

Suppl. Fig. 8. Endogenous concentrations of gibberellins, including gibberellin 1 (GA_1), GA_3, GA_4 and GA_7 in vitamin E-deficient (*vte1* and *vte4* mutants) and wild type plants of *Arabidopsis thaliana* exposed to contrasting Pi availability, including unprimed and primed plants. Data represent the mean ± SE of n=6 individuals. Significant differences between groups were tested by three-way analysis of variance (ANOVA, P<0.05). Different letters significant differences between genotypes at any given treatment (Duncan posthoc tests, P<0.05). NS, not significant.

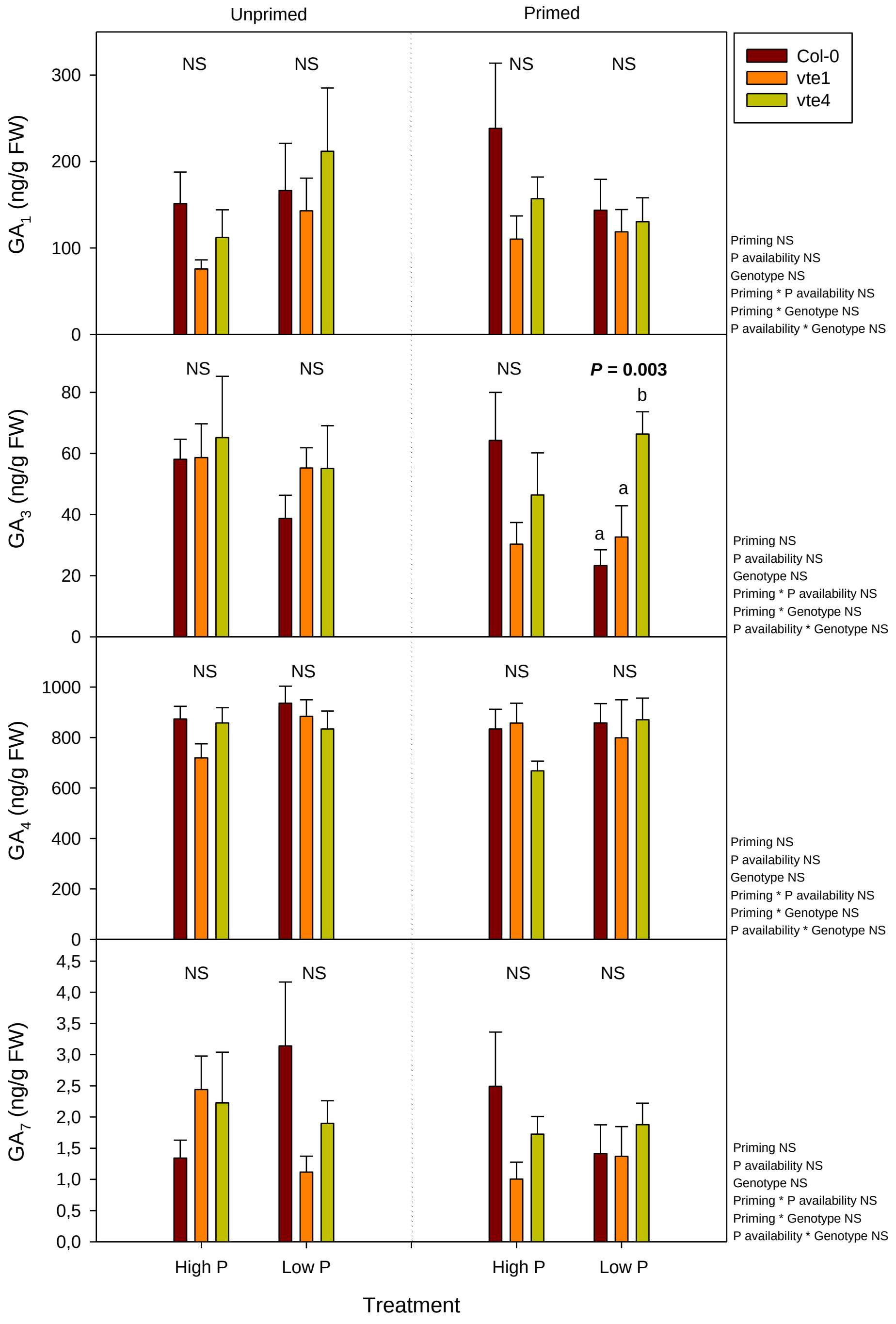

Suppl. Fig. 9. Endogenous concentrations of abscisic acid (ABA), the ethylene precursor, 1-aminocyclopropane-1-carboxylic acid (ACC) and melatonin in vitamin E-deficient (*vte1* and *vte4* mutants) and wild type plants of *Arabidopsis thaliana* exposed to contrasting Pi availability, including unprimed and primed plants. Data represent the mean ± SE of n=6 individuals. Significant differences between groups were tested by three-way analysis of variance (ANOVA, $P<0.05$). Different letters significant differences between genotypes at any given treatment (Duncan posthoc tests, $P<0.05$). NS, not significant.

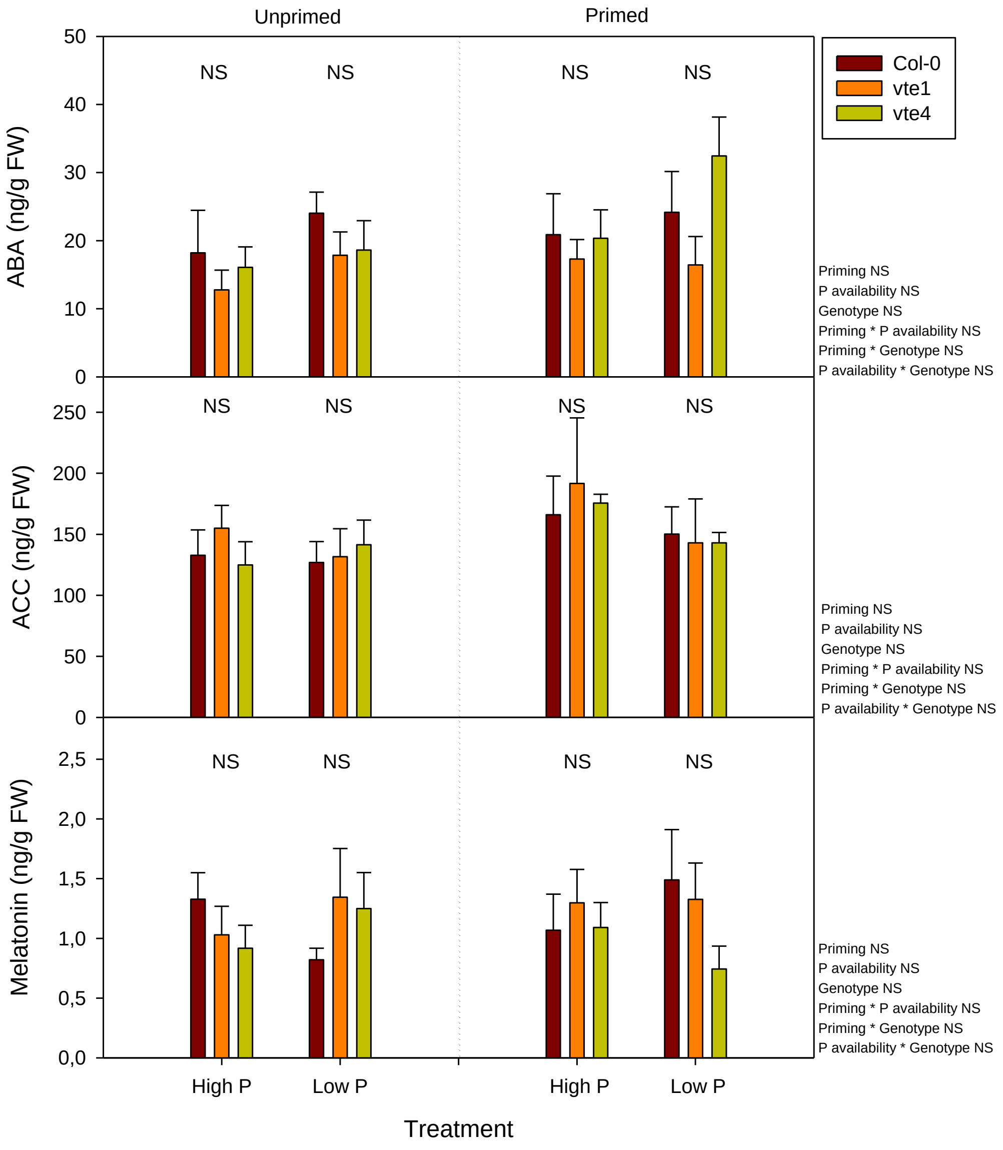

Supplement: Supplementary file 2 [file Image_1.PDF]
